# Direct Spinal Ventral Root Repair following Avulsion: Effectiveness of a New Heterologous Fibrin Sealant on Motoneuron Survival and Regeneration

**DOI:** 10.1155/2016/2932784

**Published:** 2016-08-24

**Authors:** Mateus Vidigal de Castro, Roberta Barbizan, Rui Seabra Ferreira, Benedito Barraviera, Alexandre Leite Rodrigues de Oliveira

**Affiliations:** ^1^Department of Structural and Functional Biology, Institute of Biology, University of Campinas, 13083-030 Campinas, SP, Brazil; ^2^The School of Medicine at Mucuri (FAMMUC), Federal University of Jequitinhonha and Mucuri Valleys (UFVJM), 39803-371 Teófilo Otoni, MG, Brazil; ^3^Department of Tropical Diseases, Botucatu Medical School, São Paulo State University (UNESP), 18618-000 Botucatu, SP, Brazil; ^4^Center for the Study of Venoms and Venomous Animals (CEVAP), São Paulo State University (UNESP), 18610-307 Botucatu, SP, Brazil

## Abstract

Axonal injuries at the interface between central and peripheral nervous system, such as ventral root avulsion (VRA), induce important degenerative processes, mostly resulting in neuronal and motor function loss. In the present work, we have compared two different fibrin sealants, one derived from human blood and another derived from animal blood and* Crotalus durissus terrificus* venom, as a promising treatment for this type of injury. Lewis rats were submitted to VRA (L4–L6) and had the avulsed roots reimplanted to the surface of the spinal cord, with the aid of fibrin sealant. The spinal cords were processed to evaluate neuronal survival, synaptic stability, and glial reactivity, 4 and 12 weeks after lesion. Sciatic nerves were processed to investigate Schwann cell activity by p75^NTR^ expression (4 weeks after surgery) and to count myelinated axons and morphometric evaluation (12 weeks after surgery). Walking track test was used to evaluate gait recovery, up to 12 weeks. The results indicate that both fibrin sealants are similarly efficient. However, the snake-derived fibrin glue is a potentially safer alternative for being a biological and biodegradable product which does not contain human blood derivatives. Therefore, the venom glue can be a useful tool for the scientific community due to its advantages and variety of applications.

## 1. Introduction

Spinal roots injuries constitute an important medical problem and usually affect the brachial plexus in consequence of high energy trauma, causing motor, sensibility, and autonomic loss [1–5]. The brachial plexus injury is extremely debilitating for the patient because, besides significant upper limb function loss, it causes an everyday tasks limitation [6], which can result in unemployment, depression, and in some cases even suicide [6]. Such medical problem is typically associated with young patients following motorcycle and radical sports accidents [1].

Root avulsion results in interruption of neurotrophic factors flow towards severed motoneurons, vascular trauma, and excitotoxicity, leading to drastic 80% death during the following 2 weeks after injury [7–9]. Together with neurodegeneration, synaptic changes in the spinal cord microenvironment cause expressive reduction of complexity and decreased number of presynaptic boutons apposing to surviving motoneurons [10–15]. Astroglial and microglial reactivity [16], together with the presence of meningeal fibroblasts [17], contribute to the formation of the so-called glial scar, minimizing chances of target organ reinnervation, culminating with an irreversible state of paralysis [15].

Currently, the repair of spinal root injuries is a highly delicate challenge, being particularly uncertain because of the risk of additional damage to the spinal cord, since the procedure is based on pia mater suturing [1]. Nevertheless, the surgical reimplantation of injured ventral roots has been shown to be efficient and neuroprotective [18]. In patients, it may be considered promising treatment to partially recover motor and autonomic functions, as well as reduce untreatable neuropathic pain [1, 19].

Due to the fact that direct pial suture is not always possible because of tissue loss, alternative apposition and fixation of avulsed roots with surgical adhesives have been proposed. Among available sealants, fibrin glue is biocompatible and permissive to axonal regeneration. They are produced with constituents of human blood [20] that, when combined, form a fibrin bioactive matrix with adhesive and hemostatic proprieties mimicking the final steps of the clotting cascade [21]. The use of such sealants on clinical practice is already known and widely discussed. Among many different applications, neurosurgery is of particular interest, due to the specific need of repair stability and absence of side effects [22].

In order to avoid potential adverse effects of using human derived components, such as disease transmission, researchers from CEVAP, UNESP, Brazil, produced a new fibrin sealant, in which the thrombin fraction is substituted by a protein from* Crotalus durissus terrificus* venom. The venom has thrombin-like fraction demonstrated by Nahas et al. [23] and isolated by Raw et al. [24]. The thrombin-like fraction has the ability to transform the fibrinogen directly into fibrin, forming a stable clot [25–29]. Also, bubaline fibrinogen substitutes the human counterpart [26]. As an alternative to commercial glues, such new biological product is more flexible in terms of formulation matching specific surgical needs, what can be an advantage to the commercial equivalent. Additionally, its production costs are lower, allowing broader application [26, 29].

Nevertheless, to date there is no comparative study, indicating that CEVAP's sealant performs equally well to currently marketed similars. In this regard, the present study demonstrates that root implantation, on the site of injury, can be carried out in a reproducible way with both commercial and nonhuman derived sealants.

## 2. Material and Methods 

### 2.1. Experimental Animals

The present study was carried out using ten-week-old adults female Lewis rats (LEW/HsdUnib) weighting around 200 g. The animals were obtained from the Multidisciplinary Center for Biologic Research (CEMIB) at the University of Campinas (UNICAMP), São Paulo, Brazil. The experiments were conducted according to the ethical standards for animal experimentation and were approved by the Ethical Committee for Animal Use (CEUA, UNICAMP, protocol 3064-1). Animals were subjected to unilateral ventral root avulsion and reimplantation at the lumbar intumescence (L4, L5, and L6 right side; *n* = 5 for each experimental group and technique). The contralateral roots to avulsion were used as the control for result analysis. The following experimental groups were constituted: (I) ventral root avulsion alone; (II) avulsion and reimplantation of ventral roots with fibrin sealant produced from the* Crotalus durissus terrificus* venom (CEVAP); (III) avulsion and reimplantation of ventral roots with commercial fibrin sealant Tissucol® (Baxter AG, Vienna, Austria).

### 2.2. Ventral Root Avulsion (VRA)

The rats were anesthetized by a combination of xylazine chlorhydrate (Anasedan®, 10 mg/Kg, Sespo Indústria e Comércio, Paulínia/SP, Brazil) and ketamine hydrochloride (Dopalen®, 50 mg/Kg, Sespo Indústria e Comércio, Paulínia/SP, Brazil) and subjected to unilateral avulsion of the lumbar ventral roots as previously described [2, 30–34]. Unilateral avulsion was performed at the L4–L6 lumbar ventral roots after unilateral laminectomy (right side). A longitudinal incision was made to open the dural sac, and the denticulate ligament was dissected. Finally, the ventral and dorsal roots were carefully separated so that the ventral roots associated with the lumbar intumescence could be identified and avulsed with fine forceps (Dumont®, Switzerland, Part number 11242-40). After lesioning, the roots and spinal cord were returned to their original position, and the musculature, fascia, and skin were sutured in layers. Chlorhydrate of tramadol (Germed Farmacêutica Ltda, Hortolândia/SP, Brazil) was administrated by gavage after the surgical procedures (20 mg/kg) and 2.5 mg/day soluble in water during 5 days. The animals were housed under a 12-hour light/dark cycle and controlled temperature (23°C), with free access to food and water.

### 2.3. Roots Reimplantation and Fibrin Sealants

In the VRA + sealant groups, the roots were replaced at the exact point of detachment, on the ventral surface of the lumbar spinal cord at the avulsion site with the aid of fine forceps. The fibrin sealant derived from snake venom was kindly supplied by the Center for the Study of Venoms and Venomous Animals (CEVAP) of UNESP; its constituents and instructions for use are stated in the respective patents (registration numbers BR1020140114327 and BR1020140114360). This sealant was composed of three separate solutions and homogenized immediately before use in a total final volume of 6 mL: (1) fibrinogen derived from bubaline blood (3 mL), (2) calcium chloride (2 mL), and (3) a thrombin-like enriched fraction (1 mL) [25–29].

During surgical repair of the avulsed roots, the first two components were applied and the avulsed roots were returned to their original sites. The third component was then added for polymerization. The reimplanted roots were then gently pulled from the spinal cord, and the stability of the fixation was observed to evaluate the success of the repair, while the commercial fibrin sealant utilized was Tissucol (Baxter AG, Vienna, Austria). This sealant is a lyophilized concentrate of human proteins and composed by 4 components: (1) lyophilized fibrinogen; (2) aprotinin solution; (3) lyophilized thrombin; and (4) calcium chloride solution. At the time of use, the components were previously thawed, reconstituted, mixed, and applied following the manufacturer instructions.

### 2.4. Functional Analysis

For the gait recovery analysis, the CatWalk system was used (CatWalk, Noldus Inc., Wageningen, Netherlands; http://www.noldus.com/animal-behavior-research/products/catwalk). In this setup, the animal crosses a walkway with an illuminated glass floor. A green LED illuminates the long edge of the floor so that the light highlights only the places where the animal's paws touch the glass surface. By illuminating the footprints, the plantar surfaces were captured by a high-speed video camera (Fujinon DF6H-1B) equipped with a wide-angle lens (8.5 mm, Fujicon Corp., China) positioned underneath the walkway. The paw prints are automatically recorded and classified by the software and were recorded before and after the VRA. Postoperative CatWalk data were collected weekly for 12 weeks. The peroneal functional index (PFI) was calculated as the distance between the third toe and hind limb pads (print length) and the distance between the first and fifth toes (print width). Measurements of these parameters were obtained from the right (lesioned) and left (unlesioned) paw prints, and the values were calculated using the following formula by Bain et al. [35]: PFI = 174.9 × ((EPL − NPL)/NPL)) + 80.3 × ((ETS − NTS)) – 13.4, where N is normal or nonoperated side; E is experimental or operated side; PL is print length; TS is total toe spread or distance between first and fifth toe. The pressure exerted on the platform by individual paws was also evaluated. The CatWalk data from each day of evaluation were expressed as an ipsi/contralateral ratio.

### 2.5. Specimen Preparation

The animals were anaesthetized with an overdose of a combination of xylazine chlorhydrate (Anasedan, 10 mg/Kg, Sespo Indústria e Comércio, Paulínia/SP, Brazil) and ketamine hydrochloride (Dopalen, 50 mg/Kg, Sespo Indústria e Comércio, Paulínia/SP, Brazil) and the vascular system was rinsed by transcardial perfusion with 0.1 M saline phosphate buffer (pH 7.38) followed by fixative solution. For myelin fibers counting and morphometry (sciatic nerve regeneration analysis), the rats were killed and fixed by transcardial perfusion with 2,5% glutaraldehyde and 1% paraformaldehyde in 0,1 M phosphate buffer (pH 7,38). A 10 mm midthigh segment of the sciatic nerve (ipsi and contralateral) was exposed, dissected, and stored in the same fixative solution, for 24 hours at 22°C. The fragments were washed with 0.1 M saline phosphate buffer (pH 7.38) and postfixed for 3 hours in osmium tetroxide solution 1% diluted in phosphate buffer (pH 7.38). After the postfixation the fragments were washed in distilled water and dehydrated through ethanol series and acetone (for 60 minutes) and embedded in Durcupan ACM (Fluka, Steinheim, Switzerland). The specimens were trimmed and semithin sections (0.5 *μ*m) were obtained in ultramicrotome (Leica Ultracut UCT Ultramicrotome). For neuronal survival counting and immunohistochemical evaluation (*n* = 5 for each group) the rats were killed and fixed by transcardial perfusion with 10% formaldehyde in 0.1 M phosphate buffer (pH 7.38). The lumbar intumescence and sciatic nerves (ipsi and contralateral) were exposed, dissected out, postfixed for 24 hours at 22°C in the same fixative solution, and then washed in 0.1 M phosphate buffer (pH 7.38) and subjected to 10, 20, and 30% sucrose in 0.1 M PB for 24 h each, before freezing. The specimens were embedded in Tissue-Tek® O.C.T. (Sakura Finetek USA, Inc., Torrance, CA USA) and frozen at −33° to −40°C. Transverse sections (12 *μ*m thickness) of spinal cords and longitudinal sections (12 *μ*m thickness) of sciatic nerves were obtained in cryostat (Microm HM 525®, MICROM International GmbH-Walldorf, Germany) and transferred to gelatin-coated slides and dried at room temperature for 30 min before being stored at −20°C until the moment of use.

### 2.6. Counting of Myelinated Axons and Morphometric Analysis

The sciatic nerves were processed for myelinated axon counting and morphometric evaluation by transverse semithin sections (0.5 *μ*m thick), stained in Sudan black (0.7% in 70% alcohol) solution, and examined using a bright field microscope Leica CTR 6500® (Leica Microsystems CMS GmbH) and Adobe Photoshop CS5® (Adobe Systems) software. The analyses were performed by sampling at least 30% of each nerve cross section (magnification of 1,000x), generating approximately 12 images per animal. Sampling bias was avoided by spreading the micrographs systematically over the entire cross section, according to the procedure proposed by Mayhew and Sharma [36]. The images were used for counting the total number of myelinated axons in each specimen. For the morphometric analysis, two sampled fields in each nerve (magnification of 1,000x) were utilized. The images were converted to black and white, so that any background artefacts would not be measured, including fibers that were partly captured. The myelinated axons were identified and the measurements were carried out automatically, after calibration. The measurements provided were myelin sheath's area, perimeter, and smaller diameter of each myelinated fiber. These values were used to calculate the myelinated axons diameter, myelin thickness (fiber diameter − axon diameter/2) and “*g*” ratio (axon diameter/fiber diameter). The data are represented as the mean ± standard error (SE) for each group.

### 2.7. Motoneuron Survival

Transverse cryostat sections of the spinal cords were stained in aqueous toluidine blue (1 mg/100 mL) for 3–5 minutes at room temperature. The sections were washed with distilled water, dehydrated in crescent series of alcohol and xylene, and mounted with Entellan® (Merck KGaA, Darmstadt, Germany). The motoneurons present in the lateral motor nucleus of the anterior horn on the ipsilateral side (injured) and contralateral side (not injured) were identified (based on their morphology, size, and location in the dorsolateral lamina IX) and counted in alternate sections of each specimen in about 12 sections with a 192 *μ*m space between them. Only the cells with visible nucleus and nucleolus were counted. The percentage of surviving cells was analyzed by the ratio of absolute numbers of motoneurons, counted per section, on the lesioned and nonlesioned sides, respectively, and multiplying the result by 100. Abercrombie's formula [37] was used to correct the duplicate counting of neurons: *N* = *nt*/(*t* + *d*), where “*N*” is the corrected number of counted neurons, “*n*” is the number of counted cells, “*t*” is the thickness of the sections, and “*d*” is the average diameter of neuronal nuclei. As the difference in the size affects the cells number significantly, “*d*” value was calculated for each experimental group (ipsilateral and contralateral) specifically. In order to this, the nuclear diameter of 15 randomly chosen motoneurons from each group was measured (ImageTool software, version 3.00, The University of Texas Health Science Center, TX) and the mean value calculated. The data are represented as the mean ± standard error (SE) for each group.

### 2.8. Immunofluorescence

Transverse cryostat sections of the spinal cords and longitudinal cryostat sections of the sciatic nerves were acclimatized, washed in 0.1 M phosphate buffer (pH 7.38, 3 times of 5 minutes each), and incubated for 45 min in a 3% BSA (bovine serum albumin) solution in 0.1 M phosphate buffer (pH 7.38) followed by incubation with the primary antibodies (Table 1). The primary antibodies were diluted in a solution containing 1% BSA (bovine serum albumin) and 2% Triton in 0.1 M phosphate buffer (pH 7.38). All sections were incubated for 4 hours at room temperature in a moist chamber. After rinsing in PB, the sections were incubated with a Cy3-conjugated secondary antiserum (1 : 250, Jackson Immunoresearch, West Grove, PA, USA) and diluted in 1% BSA and 0.2% Triton in 0.1 M PB (pH 7.38) for 45 minutes in a moist chamber at room temperature. The sections were then rinsed in 0.1 M PB (pH 7.38) and mounted in a mixture of glycerol/PBS (3 : 1) and observed in fluorescence microscope Leica DM5500B microscope coupled with a Leica DFC345 FX camera (Leica Microsystems CMS GmbH) utilizing rhodamine filters (CY3). For quantitative measurements, three representative images of the spinal cord (L4–L6 at lamina IX, ventral horn) and sciatic nerve from each animal were captured at a final magnification of 200x. For the quantification, the integrated density of pixels, which represents the intensity of labeling, was measured utilizing the ImageJ 1.33u (National Institutes of Health, USA) software. For the analysis of anti-glial fibrillary acidic protein (GFAP) and anti-ionized calcium binding adaptor molecule 1 (Iba1) antibodies, the integrated density of pixels was measured in the lamina IX ventrolateral, as described by Oliveira et al. [38] and Freria et al. [39]. For analysis of synaptophysin immunolabeling, the integrated density of pixels was systematically measured in eight representative areas surrounding each motoneuron located at lamina IX, in the anterior horn of the spinal cord, according to Oliveira et al. [38]. The proportion of integrated density of pixels was calculated for each animal and then as the mean value for each spinal cord. The data are represented as the mean ± standard error (SE) for each group.

### 2.9. Statistical Analysis

Statistical analysis was performed with GraphPad Prism 4.0 software. The neuronal survival, myelinated fiber counting, and immunofluorescence data were evaluated via “one-way analysis of variance.” Data from the functional analysis (walking track test) and morphometry of myelinated fibers were evaluated via “two-way analysis of variance.” “Bonferroni Multiple Comparison Test” and “Newman-Keuls Multiple Comparison Test” were used to identify intergroup differences. The data are presented as the mean ± standard error (SE) and the differences between groups were considered significant when the *P* value was >0.05 (*∗*), >0.01 (*∗∗*), and >0.001 (*∗∗∗*).

## 3. Results

### 3.1. Neuroprotective Effects after Root Reimplantation

**Figure 1:**
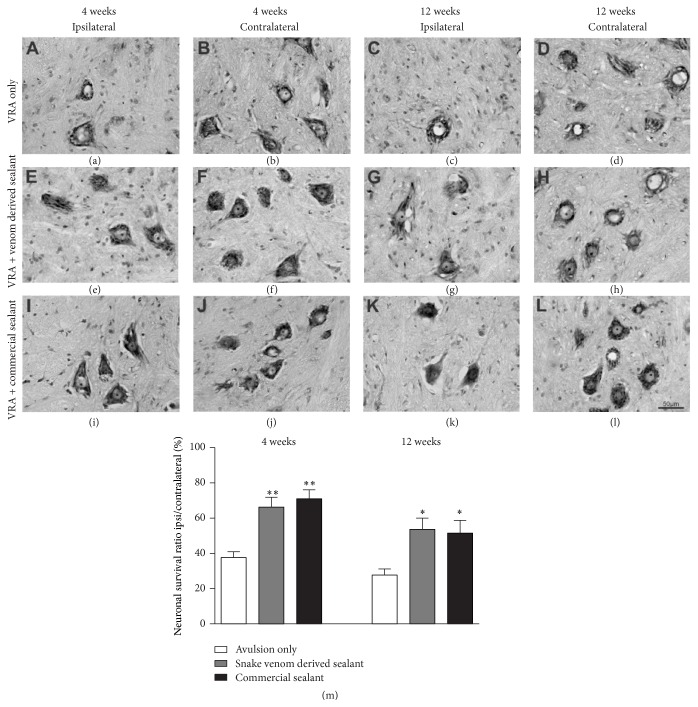
Nissl-stained spinal cord transverse sections at lamina IX illustrating the neuroprotective effect of root reimplantation on motoneurons, 4 and 12 weeks following ventral root avulsion and repair. Note the decreased number of motoneurons, ipsilateral to the lesion. Also, observe the improvement of neuronal survival in the reimplanted groups. (a-b, e-f, and i-j) Ipsilateral and contralateral sides, 4 weeks after lesion. (c-d, g-h, and k-l) Ipsilateral and contralateral sides, 12 weeks after lesion. (m) Percentage of neuronal survival 4 and 12 weeks following ventral root avulsion and reimplantation. The reimplanted groups displayed a significantly increased neuronal survival, 4 and 12 weeks after lesion. Scale bar = 50 *μ*m. Mean ± SE. ^*∗*^ *p* < 0.05; ^*∗∗*^ *p* < 0.01.

Neuronal survival after VRA and reimplantation was evaluated by the counting of motoneurons present at lamina IX of the ventral horn, in the ipsilateral side as compared to contralateral side, 4 and 12 weeks after lesion (Figure 1). No statistical differences regarding motoneuron numbers in the contralateral side, in the different experimental conditions, were observed. Based on that, all results were expressed as percentage of surviving motoneurons in comparison to the contralateral side. Four weeks after lesion, a significant loss of motoneurons could be observed in the VRA group, due to the injury (Figure 1(a)). Contrarily, the reimplanted groups displayed statistically higher number of surviving motoneurons (Figures 1(e), 1(i), and 1(m)). Also, no differences between the reimplanted groups were observed (*VRA Only:  *37.59 ± 3.40;* VRA + Venom Glue*: 66.27 ± 5.63;* VRA + Commercial Glue*: 70.93 ± 5.21; mean ipsi/contralateral ratio ± SE;* F*_2,12_ = 13.86;* P* value = 0.0008; *N* = 5 per group). Such neuroprotective effect remained up to 12 weeks after lesion, when the neuronal survival in the reimplanted groups was statistically superior to the VRA Only (Figures 1(c), 1(g), and 1(k)). Once again, no differences between the reimplanted groups were observed (*VRA Only*: 27.75 ± 3.42;* VRA + Venom Glue*: 53.64 ± 6.43;* VRA + Commercial Glue*: 51.57 ± 7.24; mean ipsi/contralateral ratio ± SE;* F*_2,12_ = 5.883;* P* value = 0.016; *N* = 5 per group).

### 3.2. Reduction of Synaptic Elimination after Reimplantation

**Figure 2:**
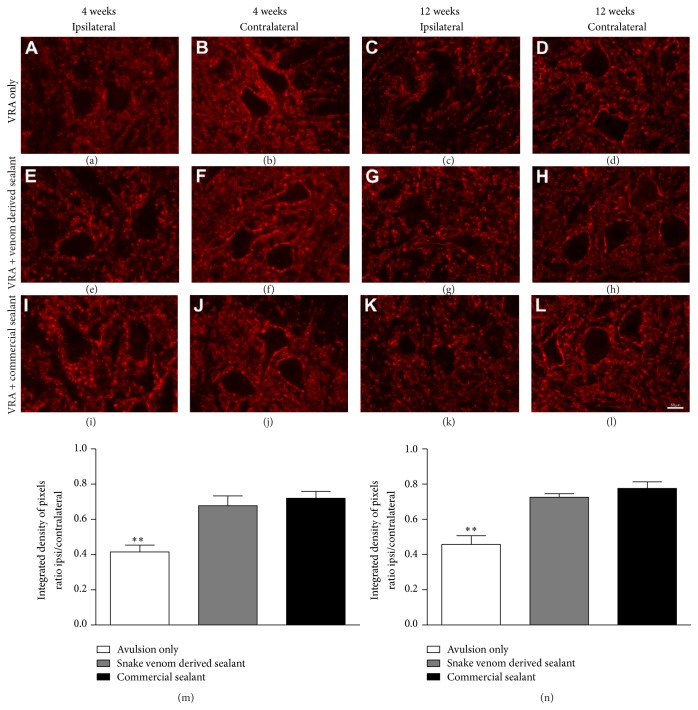
Immunohistochemical analysis of the spinal cord ventral horn stained with antisynaptophysin, 4 and 12 weeks, following ventral root avulsion and repair. A significant preservation of synaptophysin immunoreactivity was observed in both reimplanted groups. (a-b, e-f, and i-j) Ipsilateral and contralateral sides, 4 weeks after lesion. (c-d, g-h, and k-l) Ipsilateral and contralateral sides, 12 weeks after lesion. (m) Synaptic covering, obtained by the ratio IL/CL (ipsi/contralateral sides) of the integrated density of pixels at lamina IX, 4 weeks after lesion. (n) Synaptic covering, obtained by the ratio IL/CL (ipsi/contralateral sides) of the integrated density of pixels at lamina IX, 4 weeks after lesion. Observe the significant reduction of synaptic elimination in both groups repaired with fibrin sealant. Scale bar = 50 *μ*m. Mean ± SE. ^*∗∗*^ *p* < 0.01.

Synaptic activity changes after root avulsion and reimplantation were evaluated in the sciatic motor nucleus in the ventral horn of the spinal cord, by synaptophysin immunolabeling, 4 and 12 weeks after the injury (Figure 2). In both experimental survival times analyzed, the synaptophysin immunoreactivity showed higher synaptic density in the contralateral side than the ipsilateral side of avulsed animals. Such expression was drastically decreased in axotomized motoneurons surface in animals without reimplantation, indicating a significant decrease of complexity of propriospinal networks following lesion. In contrast, in the reimplanted groups, the repair resulted in preservation of synaptophysin immunoreactivity, particularly in the immediate vicinity of the motoneurons. Statistical analysis revealed no differences between the reimplanted groups (4 weeks after lesion:* VRA Only*: 0.41 ± 0.04;* VRA + Venom Glue*: 0.68 ± 0.06;* VRA + Commercial Glue*: 0.72 ± 0.04; mean ipsi/contralateral ratio ± SE;* F*_2,12_ = 13.47;* P* value = 0.0009; *N* = 5 per group); 12 weeks after lesion:* VRA Only*: 0.46 ± 0.05;* VRA + Venom Glue*: 0.72 ± 0.02;* VRA + Commercial Glue*: 0.77 ± 0.03; mean ipsi/contralateral ratio ± SE;* F*_2,6_ = 16.13;* P* value = 0.0016; *N* = 3 per group).

### 3.3. Glial Reactivity Is Not further Enhanced by Reimplantation

**Figure 3:**
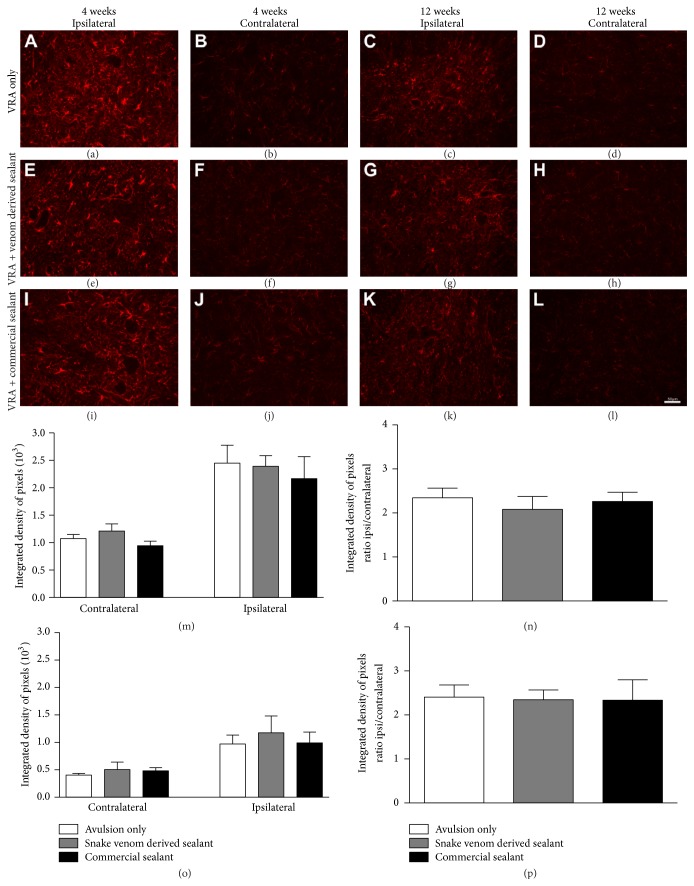
Immunohistochemical analysis of the spinal cord ventral horn stained with glial fibrillary acid protein (GFAP), 4 and 12 weeks following ventral root avulsion and repair. No significantly further increase in astrogliosis after reimplantation was observed. (a-b, e-f, and i-j) Ipsilateral and contralateral sides, 4 weeks after lesion. (c-d, g-h, and k-l) Ipsilateral and contralateral sides, 12 weeks after lesion. (m) Mean integrated density of pixels obtained ipsi and contralateral to the lesion side, 4 weeks after injury. (n) The ratio IL/CL (ipsi/contralateral sides) of the integrated density of pixels at lamina IX, 4 weeks after lesion. (o) Mean integrated density of pixels obtained ipsi and contralateral to the lesion side, 12 weeks after injury. (p) The ratio IL/CL (ipsi/contralateral sides) of the integrated density of pixels at lamina IX, 12 weeks after lesion. Scale bar = 50 *μ*m. Mean ± SE.

**Figure 4:**
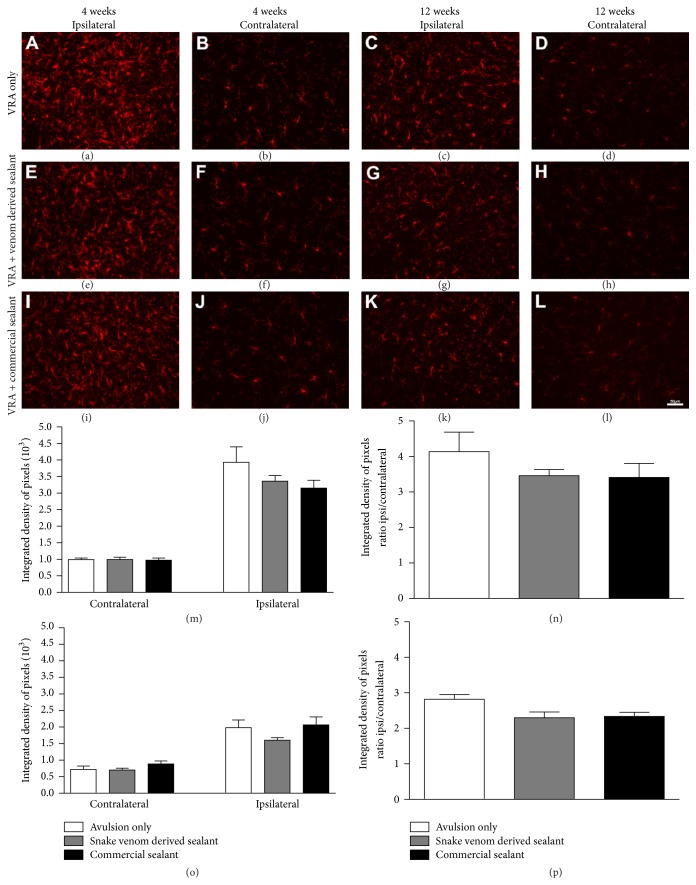
Immunohistochemical analysis of the spinal cord ventral horn stained with ionized calcium binding adaptor protein (Iba1), 4 and 12 weeks following ventral root avulsion and repair. No significantly further increase in microgliosis after reimplantation was observed. (a-b, e-f, and i-j) Ipsilateral and contralateral sides, 4 weeks after lesion. (c-d, g-h, and k-l) Ipsilateral and contralateral sides, 12 weeks after lesion. (m) Mean integrated density of pixels obtained ipsi and contralateral to the lesion side, 4 weeks after injury. (n) The ratio IL/CL (ipsi/contralateral sides) of the integrated density of pixels at lamina IX, 4 weeks after lesion. (o) Mean integrated density of pixels obtained ipsi and contralateral to the lesion side, 12 weeks after injury. (p) The ratio IL/CL (ipsi/contralateral sides) of the integrated density of pixels at lamina IX, 12 weeks after lesion. Scale bar = 50 *μ*m. Mean ± SE.

Glial reactivity after ventral root avulsion and reimplantation was evaluated in the ventral horn of the spinal cord, by immunofluorescence analysis, 4 and 12 weeks after lesion. Immunoreactivity against GFAP was used to analyze the degree of astroglial reactivity after lesion (Figure 3) while Iba-1 immunoreactivity was used to assess the degree of microglial reactivity (Figure 4). Four weeks after lesion, the immunostaining showed increased astrocyte reactivity after VRA as demonstrated by the presence of GFAP-positive activated astrocytes, particularly concentrated in the vicinity of the avulsed motoneurons. However, the astroglial reactivity in reimplanted groups was not significantly different from the VRA alone (*VRA Only*: 2.34 ± 0.22;* VRA + Venom Glue*: 2.08 ± 0.30;* VRA + Commercial Glue*: 2.26 ± 0.21, mean ratio ipsi/contralateral ± SE;* F*_2,12_ = 0,30;* P* value = 0.74; *N* = 5 per group). Similar results were observed for microglial reactivity, 4 weeks after lesion, where the immunostaining showed increased microglial reaction after VRA, as demonstrated by the presence of Iba1-positive activated microglia. In the same way, the microglial reactivity in the reimplanted groups was not significantly different from the VRA alone (*VRA Only*: 4.13 ± 0.55;* VRA + Venom Glue*: 3.46 ± 0.17;* VRA + Commercial Glue*: 3.41 ± 0.40; mean ratio ipsi/contralateral ± SE;* F*_2,12_ = 0.99;* P* value = 0.39; *N* = 5 per group). In contrast, twelve weeks after lesion, the immunostaining showed decreased glial reactivity in all experimental groups, in comparison with 4 weeks survival time. Nevertheless, no significant difference between experimental groups was observed (astroglial reactivity:* VRA Only*: 2.40 ± 0.27;* VRA + Venom Glue*: 2.34 ± 0.22;* VRA + Commercial Glue*: 2.33 ± 0.46; mean ratio ipsi/contralateral ± SE;* F*_2,6_ = 0,015;* P* value = 0.984; *N* = 3 per group; microglial reactivity:* VRA Only*: 2.82 ± 0.13;* VRA + Venom Glue*: 2.29 ± 0.16;* VRA + Commercial Glue*: 2.33 ± 0.11; mean ratio ipsi/contralateral ± SE;* F*_2,6_ = 4.639;* P* value = 0.046; *N* = 3 per group). Therefore, the glial reactivity was not significantly further increased after reimplantation in both experimental times.

### 3.4. Roots Reimplantation Affects Pan-Neurotrophin Receptor (p75^NTR^) and S100 Expression in the Regenerating Sciatic Nerve

**Figure 5:**
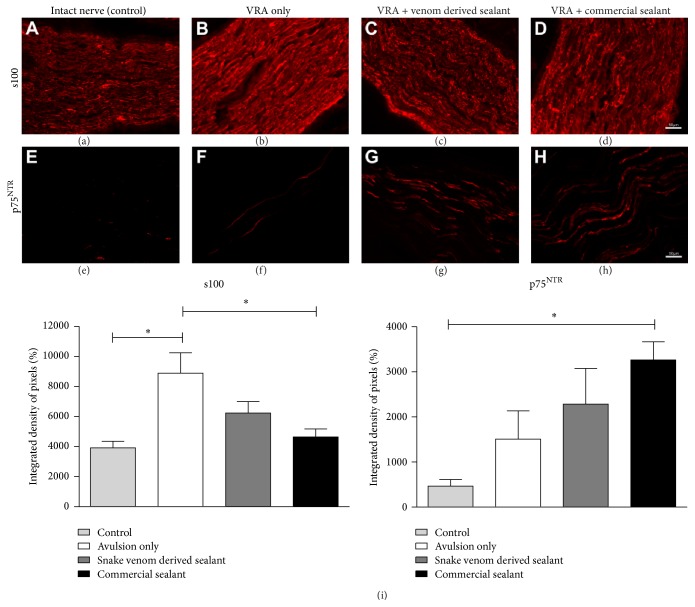
Immunolabeling anti-p75^NTR^ and S100 in sciatic nerves, 4 weeks after ventral root avulsion and repair. Increased labeling is observed in all experimental groups as compared to control. S100: (a) intact nerve; (b, c, and d) ventral root avulsion (VRA); VRA followed by reimplantation with fibrin sealant derived from snake venom; ventral root avulsion followed by reimplantation with commercial fibrin sealant, respectively. p75: (e) intact nerve; (f, g, and h) ventral root avulsion (VRA); VRA followed by reimplantation with fibrin sealant derived from snake venom; ventral root avulsion followed by reimplantation with commercial fibrin sealant, respectively. (i) Quantification of S100 and p75^NTR^ labeling by the integrated density of pixels. Scale bar = 50 *μ*m. Mean ± SE. ^*∗*^ *p* < 0.05.

Schwann cell activity (S100 labeling) and p75^NTR^ expression after ventral root avulsion and reimplantation were examined in the sciatic nerve by immunofluorescence, 4 weeks after lesion (Figure 5). p75^NTR^ is expressed on developing motoneurons and is* de novo* expressed by adult motoneurons under pathological conditions such as trauma or degeneration. In addition, roles of Schwann cells on the regenerative process include phagocytosis of axon and myelin debris derived from Wallerian degeneration, being responsible for the remyelination of axons. In this sense, it is important to identify the effects of reimplantation on p75^NTR^ and S100 expression. The immunostaining revealed an increase of such neurotrophin receptor expression after VRA as demonstrated in Figure 5, being statistically higher in the VRA + Commercial Glue group (*VRA Only*: 1,509 ± 626.5;* VRA + Venom Glue*: 2,286 ± 788.1;* VRA + Commercial Glue*: 3,266 ± 403.9;* Control*: 464.5 ± 146.1; integrated density of pixels; mean ± SE;* F*_2,12_ = 4.704;* P* value = 0.0154; *N* = 5 per group). In the same way, S100 labeling showed an increased Schwann cell activity after injury/reimplantation in all experimental groups, being more expressive in the VRA group (*VRA Only*: 8882 ± 1362;* VRA + Venom Glue*: 6237 ± 754.8;* VRA + Commercial Glue*: 4640 ± 536.3;* Control*: 3755 ± 378.1; integrated density of pixels; mean ± SE;* F*_2,12_ = 7.099;* P* value = 0.0030; *N* = 5 per group).

### 3.5. Roots Reimplantation Promoted Axonal Regeneration

**Figure 6:**
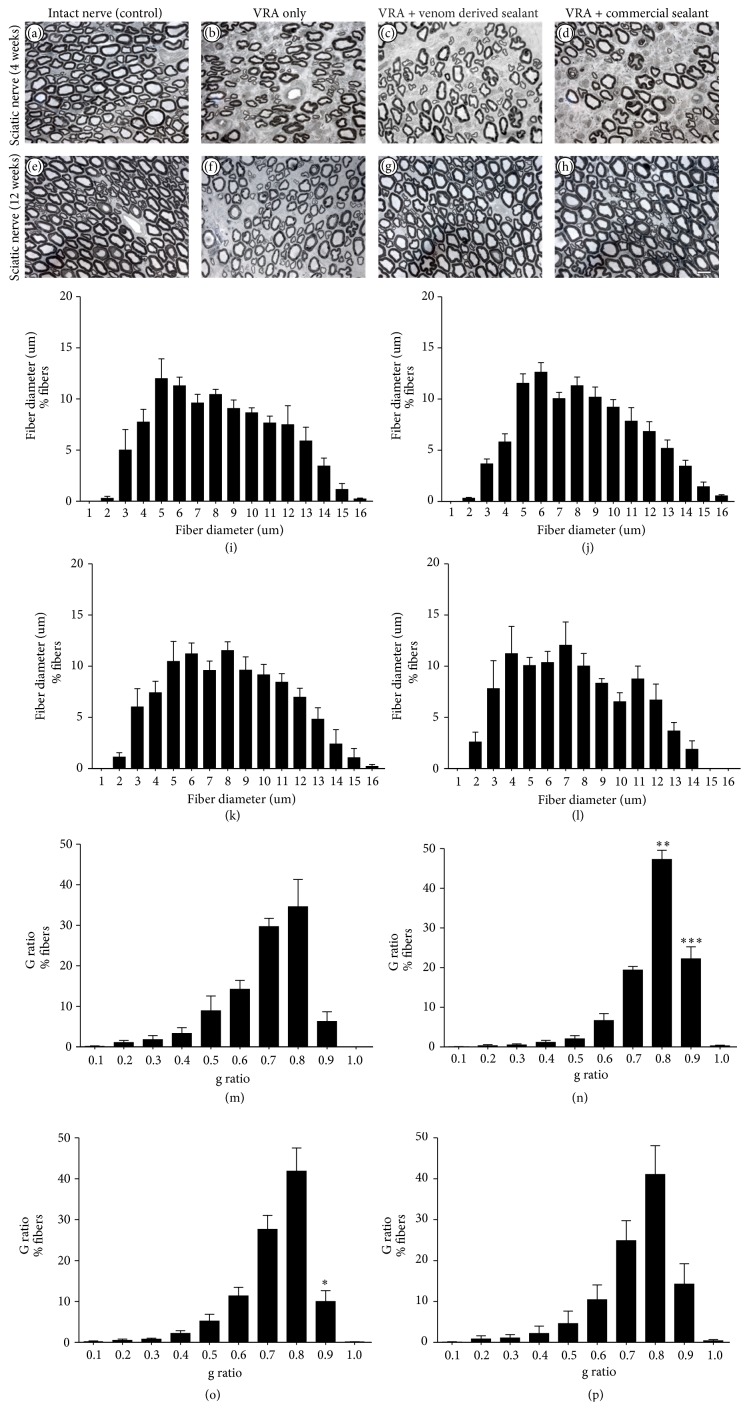
(a–d) Representative micrographs of estimated number of myelinated axons in the sciatic nerve, 4 weeks, following ventral root avulsion and repair. (e–h) Representative micrographs of estimated number of myelinated axons in the sciatic nerve, 12 weeks, following ventral root avulsion and repair. Scale bar = 10 *μ*m. (i–l) Frequency distribution of fiber diameter of regenerated fibers, 12 weeks, following ventral root avulsion and repair. (m–p) Frequency distribution of *g* ratio of regenerated fibers, 12 weeks, following ventral root avulsion and repair. ^*∗*^ *p* < 0.05; ^*∗∗*^ *p* < 0.01; ^*∗∗∗*^ *p* < 0.001.

The number of myelinated axons present in the sciatic nerve was estimated based on the total area of the nerve and on the fiber number in the evaluated fields. After 12 weeks after injury (Figure 6), the number of myelinated axons was higher in reimplanted groups when compared to VRA group, showing the capacity of regeneration of motor axons after reimplantation, allowing growth towards the target muscles (VRA group 6,219 ± 231.4; VRA + Snake Venom Sealant group 7,177 ± 486.7; VRA + Commercial Sealant group 7,508 ± 151.5; Contralateral group 8,093 ± 305.1 mean ± SE). No significant differences between the reimplanted groups were observed. In addition, morphometry revealed an increase of the myelinated fibers with thin myelin sheath in the animals from VRA group (Figures 6(m)–6(p)). In the reimplanted groups it was possible to notice an increased number of myelinated fibers with thicker myelin sheath in comparison to the VRA group, suggesting possible axonal regeneration. Contralateral intact sciatic nerves showed normal distribution [4, 40] and greater number of fibers.

### 3.6. Recovery of Motor Function by Reimplantation

**Figure 7:**
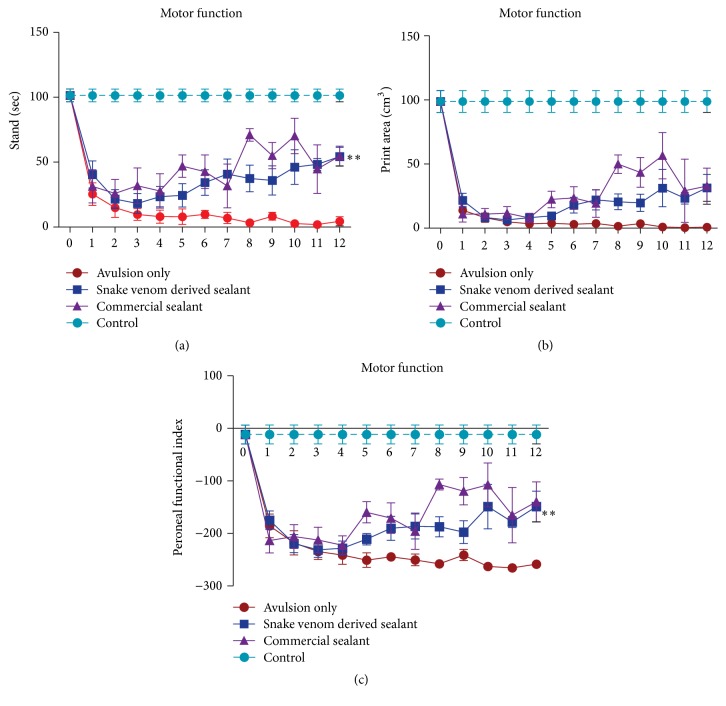
Motor function recovery up to 12 weeks after lesion (0 = preoperative). (a) Graph of the duration of contact of a paw with the glass plate in a step cycle (seconds). (b) Graph of the surface area of the complete print (cm^3^). (c) Graph of the peroneal nerve functional index. Observe the significantly improvement of motor performance in the fibrin sealant reimplanted groups compared to VRA Only from the first week after lesion until the twelfth week. A significant restoration of weight-bearing capacity following avulsion and reimplantation with the fibrin sealants is also observed. Values are expressed as the ratio IL/CL (ipsi/contralateral sides) pressure exerted by the paw on the CatWalk platform. ^*∗∗*^ *p* < 0.01.

**Figure 8:**
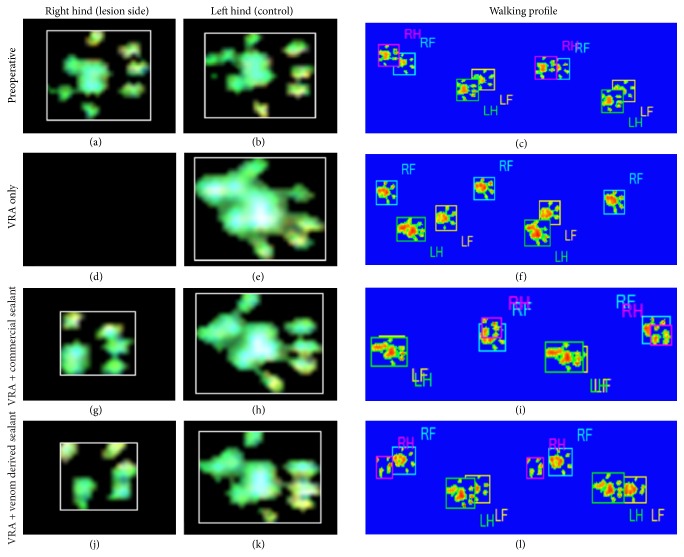
Paw print and walking profile, obtained with the CatWalk system, at 12 weeks after lesion. (a–c) Preoperative; (d–f) VRA Only; (g–i) VRA followed by reimplantation with fibrin sealant derived from snake venom; (j–l) ventral root avulsion followed by reimplantation with commercial fibrin sealant. Observe rats from reimplanted groups using the right paw, whereas the avulsed rat without root repair cannot control the lesioned limb. A significant restoration of weight-bearing capacity following avulsion and reimplantation with the fibrin sealants was also observed.

The recovery of the functional recovery was evaluated using the CatWalk system (Noldus Inc., Netherlands, Figure 7), from the 1st until the 12th week after injury. Postoperative assessments of peroneal function were performed weekly. The preoperative peroneal functional index mean values did not significantly differ between groups. At the first postoperative week, all groups have shown drastic decrease in mean PFI, indicating functional loss. Nevertheless, at the 12th postoperative week, the reimplanted groups presented significantly higher mean PFI compared to the VRA group, according to the formula described by Bain et al. [35]. Statistical analysis revealed no differences between the reimplanted groups. Furthermore, these results are consistent with the footprint paw pressure data indicating that the reimplanted groups performed better in supporting their body weight on the injured limb (Figure 8; see Supplementary Videos 1–4 in Supplementary Material available online at http://dx.doi.org/10.1155/2016/2932784).

## 4. Discussion

Development of new strategies for treating spinal root avulsion is of great medical relevance. Experimentally, it is possible to mimic human brachial plexus lesions by disconnecting ventral roots from the surface of the spinal cord. The result is ipsilateral limb paralysis, with absence of spontaneous recovery. We have previously suggested a root reimplantation method, by apposing avulsed roots to the lesion site, stabilizing the repair by the application of a fibrin scaffold, derived from nonhuman blood [2]. Here we compare such restoration performance and functional recovery with a commercial fibrin sealant Tissucol (Baxter AG, Vienna, Austria), analyzing data 4 and 12 weeks after surgery.

Ventral root avulsion results in significant spinal motoneuron degeneration (MN), combined with glial reactivity and synaptic circuits shrinkage [41]. It is known that such proximal lesion induces loss of about 80% of axotomized motoneurons during the first 2 weeks after the injury [7, 42, 43]. In this aspect, the present data allow concluding that reimplantation of avulsed roots is neuroprotective by itself. After 4 weeks of injury a lower rate of neuronal death was observed, regardless the fibrin glue employed for the root repair. Further, reimplanting neuroprotective effects were long-lasting, as they were present at the same levels up to 12 weeks after injury. These findings are in line with Hallin and contributors [44] that suggested motoneuron survival after root repair is based on neurotrophic factor production by glial cells at the CNS/PNS interface. This has also been connected to the breakage of the blood-brain barrier, allowing neurotrophic factor producing immune cells to enter spinal cord gray matter environment [45–47]. Nevertheless, Eggers et al. [48] demonstrated that roots repair is unable to completely hampers neurodegeneration.

Besides neuronal death, a hallmark of VRA is the reduction of presynaptic terminals to motoneurons. Such process decreases or even abolishes synaptic transmission [11–13]. In agreement with the literature, we observed extensive synaptic loss after avulsion without reimplantation. Importantly, the groups that were subjected to root repair showed significant preservation of synapses when compared to the avulsion alone. Therefore, it is possible that early reparation of lesion stabilizes spinal circuits in an inhibition/excitation proportion compatible with preservation of regenerating neurons.

Coupled with the above discussed synaptic changes, glial cells become reactive following VRA. Such reactive gliosis is characterized by hypertrophy of the cell body and processes of astrocytes and microglial hyperplasia [49–51]. This in turn may result in scar formation, inhibiting axonal growth [52], or lead to neurotrophic support of the axotomized neurons, resulting in regeneration. Reimplantation of ventral roots did not significantly decrease microglial and astroglial reactivity, four weeks after lesion. Ohlsson et al. [53] also did not observe differences in astroglial and microglial reaction, when comparing avulsed and reimplanted groups, at the same time point following lumbosacral ventral root avulsion injury. We believe that glial reaction following root repair did not hamper axonal sprouting, based on morphological evaluation of the sciatic nerve 12 weeks after lesion, as well as on behavioral assessment. Further, in order to achieve such gliosis downregulation, association with pharmacological/cell therapy approaches seems to be necessary.

The Wallerian degeneration processes that follow proximal axotomy became clear at the ipsilateral sciatic nerve, already at 4 weeks after injury, by the significant decrease in number of myelinated axons, combined with diminished mean values of myelin sheath, compatible to what has been described previously [7, 54]. On the contrary, 12 weeks after repair revealed improved number of fibers, indicating regeneration, regardless the fibrin sealant used. In the same fashion, myelin thickness and “*g*” ratio reach close to normal values in reimplanted groups, similar to what has been shown in the literature [40, 55]. Thus, root reimplantation has proven to be essential for the axon regrowth, allowing CNS/PNS reconnection towards target muscle fibers.

Importantly, the regeneration process followed by avulsed root repair must be evidenced via behavioral improvement by motor and gait recovery. In this sense, the use of a refined system to investigate the motor capacity can provide reliable data on animals' functional recovery [56, 57]. The mostly automatic walking track analysis, by the CatWalk system, has been recently introduced and proven to deliver highly precise data on mobility deficit/recovery detection, including the following parameters: footprint area, footprint pressure intensity, position, and footstep duration [58]. Our results demonstrated that motor function loss is evidenced immediately after injury (first week after operation), reflecting denervation of target muscles, corresponding to the territory of the sciatic nerve. Since axonal regrowth is a slow process, observation of long-term survival times is of fundamental importance. Herein we show evidence of recovery by the 8th week after repair. Best behavioral values were observed after 12 weeks following root reimplantation. Such motor recovery possibly involved plasticity of the nervous system at different levels, including motor cortex and descending pathways as well as motor unit refinement [59].

Overall, the present work demonstrates that fibrin sealant based restoration of avulsed roots is efficient and results in neuroprotection of proximally axotomized motoneurons and motor recovery. Also, our findings indicate similar regenerative performance of both biological adhesives employed, highlighting the significant potential of the nonhuman derived glue produced by CEVAP, Brazil.

## 5. Conclusions

The fibrin sealant obtained from the* Crotalus durissus terrificus* venom as well as the commercial fibrin sealant Tissucol (Baxter AG, Vienna, Austria) performed equally well for the restoration of CNS/PNS interface, following ventral root avulsion lesion. The data herein indicate that such surgical approach is neuroprotective, but a critical recovery timeframe of at least 8 weeks after surgery is necessary, reaching stable motor function by 12 weeks after operation.

## Supplementary Material

Representative gait video recording obtained from the different experimental groups during CatWalk - walking track test analysis.

## Figures and Tables

**Table 1:** Primary antibodies used for the immunofluorescence assay. Each antibody is followed by the supplier, host animal, product code, and concentration used.

Antibody	Supplier	Host animal	Product code	Concentration
Synaptophysin	Millipore	Mouse	MAB5258	1 : 1,500
GFAP	Abcam	Rabbit	AB779	1 : 250
IBA-1	Wako	Rabbit	019-19741	1 : 2,000
S-100	Dako	Rabbit	Z0311	1 : 2,000
p75^NTR^	Santa Cruz	Goat	Sc6188	1 : 250
